# Effect of weather conditions, substrate pH, biochar amendment and plant species on two plant growth-promoting microbes on vegetated roofs and facades

**DOI:** 10.1016/j.heliyon.2022.e09560

**Published:** 2022-05-31

**Authors:** Long Xie, Sari Timonen, Alan C. Gange, Kirsi Kuoppamäki, Marleena Hagner, Susanna Lehvävirta

**Affiliations:** a Department of Agricultural Sciences, University of Helsinki, PO Box 27, FI-00014, Finland; b Department of Microbiology, University of Helsinki, PO Box 56, FI-00014, Finland; c Department of Biological Sciences, Royal Holloway University of London, Egham, Surrey, TW20 0EX, UK; d Ecosystems and Environment Research Programme, Faculty of Biological and Environmental Sciences, University of Helsinki, FI-15140, Lahti, Finland; e Natural Resources Institute Finland (Luke), FI-31600, Jokioinen, Finland; f Ecosystems and Environment Research Programme, Faculty of Biological and Environmental Sciences, University of Helsinki, PO Box 65, FI-00014, Finland

**Keywords:** *Bacillus amyloliquefaciens*, Environmental factors, Microbial population, *Rhizophagus irregularis*, Vegetated roofs/facades

## Abstract

**Background:**

Vegetated building envelopes (VBEs), such as vegetated roofs and facades, are becoming more frequent in urban planning nowadays. However, harsh growing conditions restrain the application of VBEs. Plant growth-promoting microbes (PGPMs) might help ease the stresses, but first, it is necessary to investigate how to ensure their survival and growth under VBE conditions.

**Methods:**

We conducted three experiments to test the impact of various factors on the microbial populations of inoculated PGPMs in VBEs, a mycorrhizal fungus *Rhizophagus irregularis* and a bacterium *Bacillus amyloliquefaciens*. The first experiment was conducted by inoculating the two PGPMs separately in *Sedum* roof plots, and the microbial populations associated with *Poa alpina* was monitored for two consecutive years under local weather conditions. The second experiment was conducted in a laboratory testing the effect of substrate pH (substrates collected from balcony gardens) on *R. irregularis* population associated with *Trifolium repens* and *Viola tricolor*. The third experiment was conducted on a meadow roof testing the effect of biochar amendment on *R. irregularis* population associated with *Thymus serpyllum* and *Fragaria vesca*.

**Results:**

In the first experiment, *Bacillus* was found to associate with *P. alpina*, but *Rhizophagus* wasn't. Yet, the fungus induced high *Bacillus* population density in the *Rhizophagus* treated plots in the first year. In the second experiment, *Rhizophagus* abundance in *T. repens* was higher in the neutral substrate (6–6.5), while *V. tricolor* was more colonized in acidic substrate (5–5.5), suggesting an important interactive effect of substrate pH and plant species on *Rhizophagus* abundance. The third experiment suggested a negligible impact of biochar amendment on *Rhizophagus* abundance for both host plants.

**Conclusion:**

Three experiments demonstrate that PGPM inoculation on VBEs is feasible, and various factors and interactions affect the PGPM populations. This paper provides reference and inspiration for other VBE research involving substrate microbial manipulation.

## 1 Introduction

Studies of vegetated building envelopes (VBEs), including vegetated roofs and facades, are frequently motivated by the need for more green infrastructures to provide ecosystem services in cities (Shafique et al., 2018). Thus, a wealth of studies on VBEs focuses on plant community development and the ecosystem services they provide, e.g., stormwater management and urban heat island mitigation. During the recent decade, scientists have revealed that microbial communities in VBE systems are compositionally distinct compared to their ground-level counterparts (McGuire et al., 2013), and this distinction is attributed to anthropogenic manipulation, such as microbial inoculation, plant selection, and choice of substrate types and depths (Molineux et al., 2014; Hoch et al., 2019). It has also been shown that plant growth-promoting microbes (PGPMs) can improve plant growth in extreme weather conditions in VBE systems and provide enhanced ecosystem services (Henry and Frascaria-Lacoste, 2012; Molineux et al., 2017; Rumble and Gange, 2017; Wang et al., 2017; Fulthorpe et al., 2018). Yet, the results are variable and inconsistent, which might be due to unspecified environmental factors that affect the establishment, survival, and growth of PGPMs in VBEs. Thus, this paper aims to explore factors that could exert such influences.

Harvesting the desired benefits from PGPM inoculation is dependent on whether PGPMs can survive the extreme weather conditions on VBEs and form symbioses with VBE plants. It is worth trying to optimize growing conditions, e.g., manipulating substrate pH and amending substrate with aggregates that may improve the conditions for microbiota. For instance, biochar, made from biomass via pyrolysis, has been shown to increase water retention in vegetated roofs (Cao et al., 2014; Kuoppamäki et al., 2016), provide habitat for microbes to propagate (Palansooriya et al., 2019), and enhance microbial population via balanced nutrient levels (Anderson et al., 2011).

We focused on two identified PGPM species: a mycorrhizal fungus *Rhizophagus irregularis* (Blaszk, Wubet, Renker & Buscot) and a plant growth-promoting bacterium *Bacillus amyloliquefaciens* (Fukumoto). Their benefits include induced systemic resistance against pathogens, nutrient absorption, plant growth regulation through phytohormone production, and resistance to abiotic stresses (Idriss et al., 2002; Lenoir et al., 2016). *R. irregularis* is an arbuscular mycorrhizal fungus (AMF) that resides in host root tissues by forming internal structures, i.e., hyphae, arbuscules, and vesicles. They function as nutrient transportation ducts, nutrient exchange sites, and nutrient storage organs, respectively (Strack et al., 2003). *B. amyloliquefaciens* is a Gram-positive bacterial species that can be attracted by plant root exudates. A layer of *B. amyloliquefaciens* cells, known as a biofilm, will form on the root surface. The biofilm protects the host plants from underground pathogenic invasion and provides the host plants with nutrients and phytohormones (Chen et al., 2012).

The present study, consisting of three experiments, is part of a study series that investigate the use of PGPMs on VBEs in southern Finland. The inoculants and plant species were consistent throughout the VBE study series. Even though the study series revealed the effects of inoculating selected plants with *R. irregularis* and/or *B*. *amyloliquefaciens* under both controlled and rooftop conditions, there is still a knowledge gap about which factors may affect the vitality and colonizing ability of PGPMs (Xie et al., 2018, 2020). Therefore, we chose weather conditions (air temperature and precipitation), substrate pH, and biochar amendment as the three factors to investigate. Firstly, the unshaded, often thin-substrate VBE systems are susceptible to heat and drought stresses, which begs for knowledge regarding the impact of air temperature and rain intensity on microbes inoculated in substrates. Secondly, substrates in Finland are mostly acidic, ranging between 3.7-5.8 in pH (Starr et al., 1996; Mäkelä-Kurtto and Sippola, 2002). In order to support local plant species in VBEs, it is important to know how the inoculated PGPMs respond to substrate pH in VBEs (Barlow et al., 2020). Thirdly, biochar amendment in VBEs has been intensively studied, especially its function in stormwater management (Kuoppamäki and Lehvävirta, 2016; Kuoppamäki et al., 2016, 2019). Meanwhile, the overall effects of biochar on *Rhizophagus* colonization are still not clearly understood, and contradictory results have been reported under various growing conditions and experimental designs. We were intrigued to find out whether biochar amendment in VBEs also affects inoculated PGPMs. From the three experiments, we hypothesized that 1) Both *R. irregularis* and *B. amyloliquefaciens* could colonize the roof and survive the winter; 2) Substrate pH, together with plant species, would affect AMF colonization; 3) Biochar might increase AMF colonization by increasing substrate moisture and providing microbial habitat.

## 2 Materials and methods

### 2.1 Experimental layout

Three experiments, one indoors and two outdoors, were carried out between 2012 and 2017 to assess three major growing factors on PGPM inoculation, i.e., weather conditions, substrate pH, and biochar amendment (Table 1).

**Table 1:** Timeline of the three experiments.

Experiment	Factors	Establishment	Sampling times
Exp. 1: *Sedum* roof	Weather conditions	Spring 2012	June to September in 2012 and 2013 at an interval of three weeks
Exp. 2: Balcony garden^1^	Substrate pH	Spring 2017	August 2017
Exp. 3: Meadow roof	Biochar amendment	Autumn 2016	September 2017

1 Substrates were collected from the balcony gardens to cultivate indoor plants with PGPM inoculation.

#### 2.1.1 Experiment 1: Detection of PGPMs inoculated in a *Sedum* roof under local weather conditions

**Figure 1:**
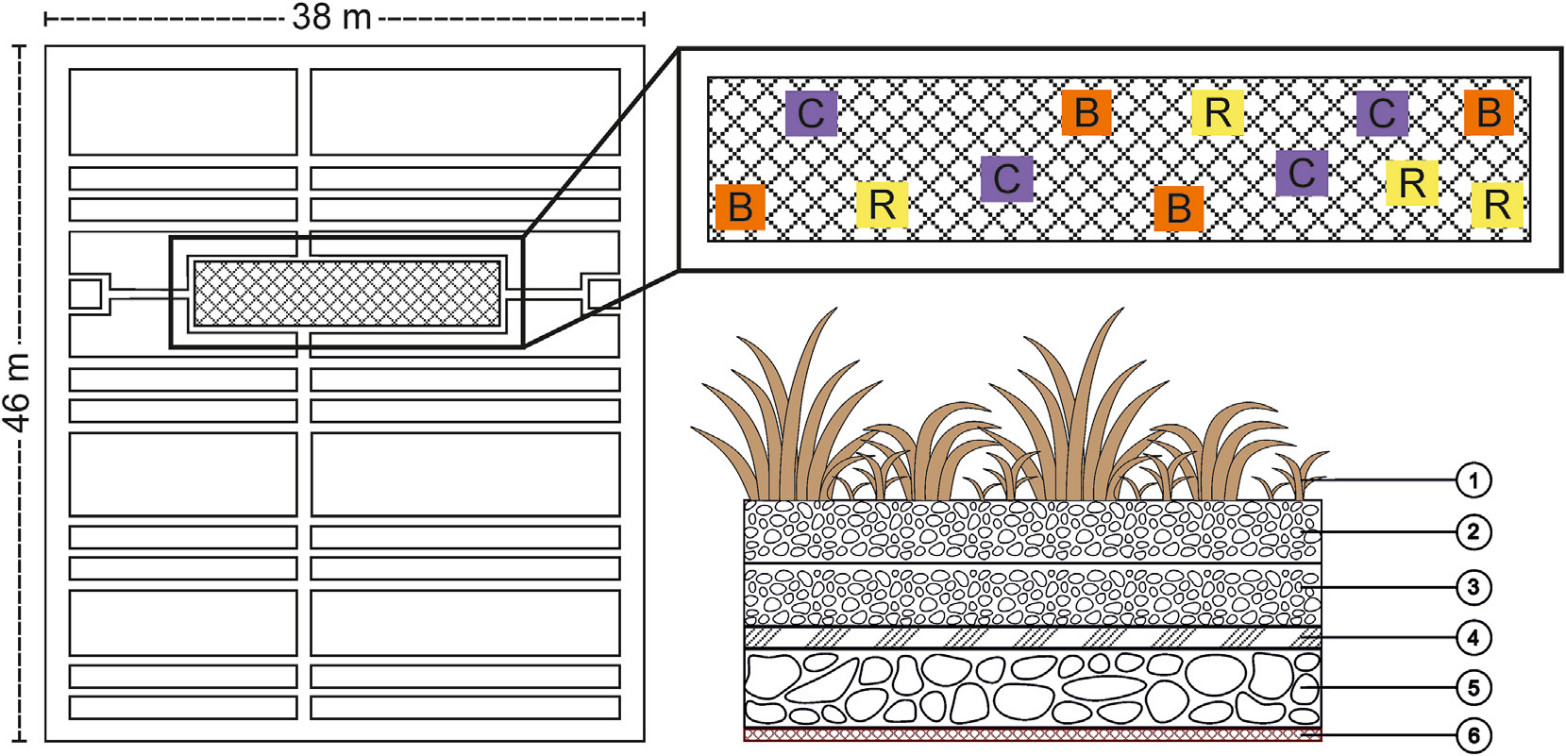
The *Sedum* roof design and layout. The picture on the left shows the layout of the whole vegetated roof. The picture on the upper right shows the layout of 12 experimental plots treated with: *R. irregularis* (R), *B. amyloliquefaciens* (B), and control (C). The picture on the lower right shows the layers of the vegetated roof. ①: plants; ②: 3 cm substrate layer came with *Sedum* mats; ③: 3 cm crushed-brick based substrate layer; ④: 1 cm filter and moisture layer; ⑤: 2.5 cm water retention and drainage layer; ⑥: 2 mm root barrier.

The vegetated roof was installed in spring 2012 for experimental purpose on an 1800 m^2^ roof of a retail store in Vantaa, Finland (60°16′47.8″N, 24°4′53.3″E). The experiment was conducted twice in the summers of 2012 and 2013. The experimental site was a 4 × 20 m vegetation patch installed with pre-grown *Sedum* mats produced by Veg Tech (Vislanda, Sweden) (Figure 1). *S. acre* and *S. telephium* were the predominant plant species on the vegetation mats. After the installation, mixed seeds (containing *Poa alpina*, *Barbarea vulgaris*, *Trifolium repens*, *Thlaspi arvense*, and *Verbascum thapsus*) from Suomen Niittysiemen Oy (Kokkola, Finland) were sown onto the experimental site as bait plants for the PGPMs.

The pre-grown mats were fixed on a 3 cm crushed-brick-based substrate layer (pH 8, organic matter 1%, P 4.3 mg/kg, and N 0.3 mg/kg). The inoculant products MYC4000 (4000 spores of *R. irregularis* strain DAOM181602/g) and Rhizocell (>10^9^ CFU endospores of *B. amyloliquefaciens* strain FZB42/g) were powdery additives purchased from Lallemand Plant Care (Castelmaurou, France). Twelve randomly located experimental plots (1.5 × 1.5 m) were treated as follows: 4 with MYC4000 (R), 4 with Rhizocell (B), and 4 with only the same amount of water as control (C) (Figure 1). 10 g of Rhizocell and 2 g of MYC4000 were applied per m^2^ by dissolving the products in tap water and irrigating the solutions onto the corresponding plots.

*P. alpina* was the only non-succulent plant species growing abundantly which produced at least five replicates from each treated plot for each sampling time. Therefore, we collected root samples and crushed-brick-based substrate samples adhering to the roots, four times per growing season (June to September in 2012 and 2013) at an interval of three weeks, to monitor microbial populations of *R. irregularis* and *B. amyloliquefaciens*. Each time, five root samples of *P. alpina* from each treated plot were collected, gently washed, mixed, and stored in 70 % ethanol. Simultaneously, the root-adhering crushed-brick-based substrate was collected from each root sample. The substrate was mixed thoroughly in tubes and stored at +4 °C. Eventually, we pooled respective samples from each treated plot, resulting in 12 root samples and 12 substrate samples for each sampling time.

The data of hourly air temperature and precipitation were retrieved from the nearest weather station (3 km) of the Finnish Meteorological Institute (https://en.ilmatieteenlaitos.fi/). According to a vegetated roof company in the USA (Columbia Green Technologies, 2014), air temperature over 23 °C and precipitation less than 6 mm per week will cause stress for plant growth on vegetated roofs. Therefore, heat stress degree hour (HSDH) above 23 °C was used to evaluate heat stress (Gu, 2016; Rayner et al., 2016). HSDH was calculated by summing up the hourly temperature difference (Ti-23 °C) for seven days before each sampling:

(1) $$HSDH\;\left(˚Ch\right)=\overset{n}{\underset{i=1}{\sum }}\left(T_{i}\;-\;23˚C\right)$$

In which “T_i_” is the recorded air temperature that was higher than 23 °C. Air temperature lower than 23 °C was not included. Total precipitation (mm) of seven days before each sampling was summed up to indicate water availability.

#### 2.1.2 Experiment 2: The effects of substrate pH ​and ​plant species on mycorrhizal abundance in balcony garden substrate

**Figure 2:**
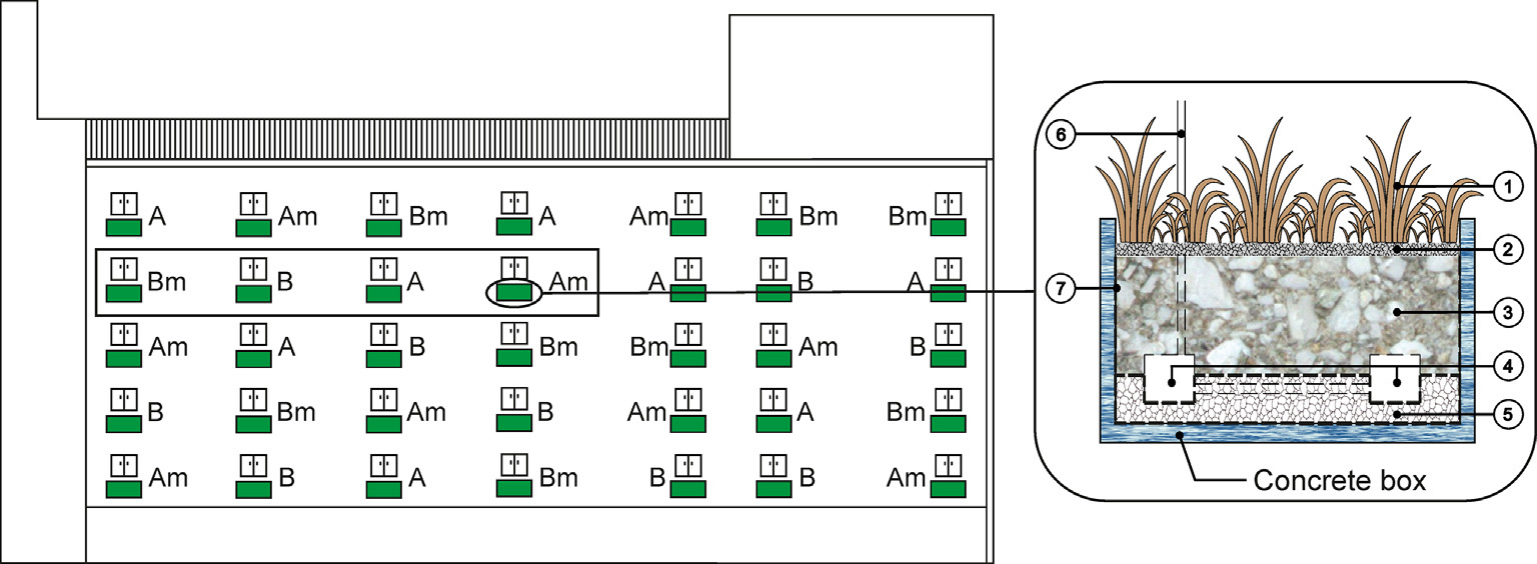
The balcony garden design and layout. The picture on the left shows the south facade of the residential building with the balcony gardens (green boxes) from which the substrates were collected (indicated in the black box). Each box was filled with one of the four substrate types, i.e., A (substrate A), B (substrate B), Am (substrate A + AMF), and Bm (substrate B + AMF). The picture on the right shows the components in the boxes. ①: plants; ②: 5 cm cover layer with gravel stone; ③: 40 cm substrate; ④: water tanks; ⑤: 10 cm drainage layer; ⑥: watering pipe linked to water tanks; ⑦: insulation and waterproofing layer.

The balcony gardens were installed on a residential building in early spring 2017 in Helsinki, Finland (60°9′18.1″N, 24°54′58.0″E). This experiment was conducted once in summer 2017. Each balcony garden is a concrete box (138.5 × 124.5 × 70.0 cm) that holds 1.2 m^3^ substrate (Figure 2). The balcony gardens were evenly and randomly assigned into four treatments according to the substrate types and mycorrhizal inoculation: substrate A (A), substrate A + AMF (Am), substrate B (B), and substrate B + AMF (Bm). Provided by Hyvinkään Tieluiska Oy (Hyvinkää, Finland), the substrate A and B were crushed-brick-based growing media that differed in pH: substrate A was acidic (pH 5–5.5), and substrate B was neutral (pH 6–6.5). The substrates had similar nutrient levels: N 50–80 mg/l, P 40–70 mg/l, and K 150–280 mg/l. AMF was added by mixing forest humus containing *R. irregularis* (10 l humus per m^3^ substrate). Pot plants of woody species (*Juniperus communis* and *Picea abies*) and climbers (*Clematis sibirica*, *Humulus lupulus*, *Hydrangea anomala*, and *Parthenocissus quinquefolia*) were planted in the balcony gardens. *J. communis* plants came from Hongiston taimisto Oy (Koski, Finland), and the rest from Terolan Taimitarha Oy (Hämeenlinna, Finland).

Due to the inaccessibility, we could not cultivate and monitor microbes in the balcony gardens. Instead, each type of substrate was collected from four balcony gardens on the fourth floor two months after the establishment using crane (Figure 2), and the collected substrates were used to grow *T. repens* and *Viola tricolor* in lab. The limitation resulted in one replicate of each substrate type, thus allowing for a preliminary assessment of the viability of *R. irregularis* in the balcony gardens.

*T. repens* and *V. tricolor* seeds purchased from Suomen Niittysiemen were grown in each of the four substrate types in laboratory conditions (130 lumens light intensity, 16/8h day/night length, 23 °C room temperature, and 35% relative humidity). The selected plants were used as bait plants to see if *R. irregularis* settled down successfully in the balcony gardens. For each plant species and substrate type, six individual plants were cultivated in separate pots for two months before sampling as biological replicates.

#### 2.1.3 Experiment 3: Effect of biochar amendment on mycorrhizal abundance in a meadow roof

**Figure 3:**
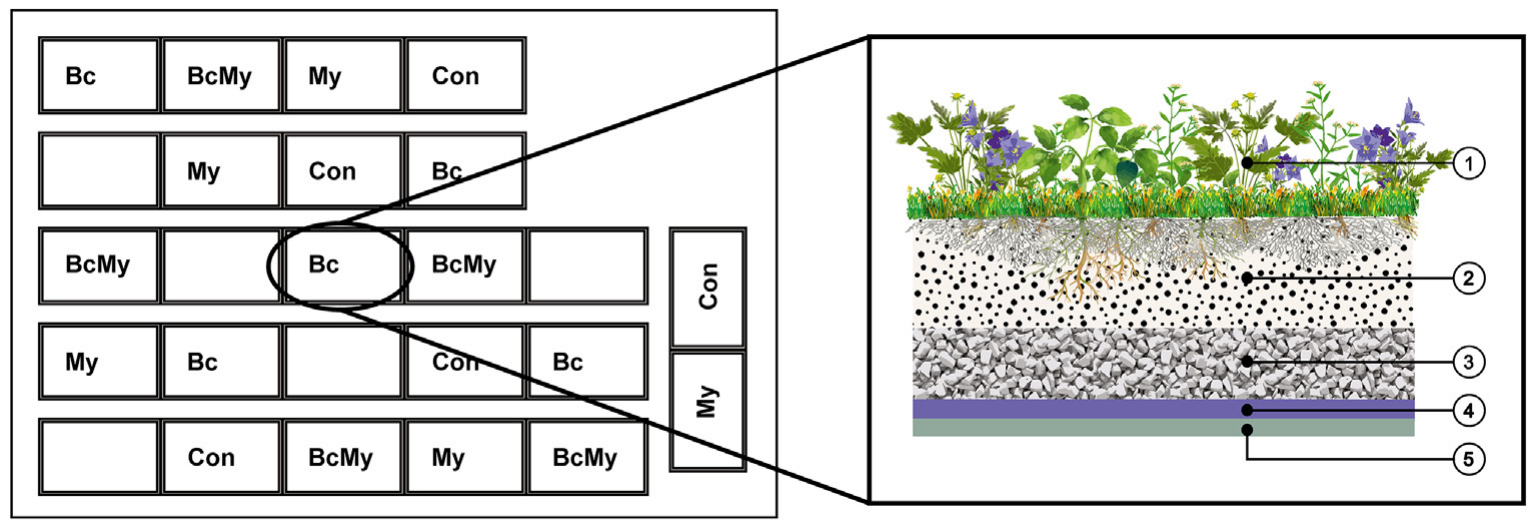
The meadow roof design and layout. The picture on the left shows the test boxes on the roof containing 4 substrate types: Con (lightweight crushed concrete 70%, compost 25%, pinewood chips 5%); Bc (lightweight crushed concrete 50%, biochar 20%, compost 25%, pinewood chips 5%); My (Con with mycorrhiza); and BcMy (Bc with mycorrhiza). The picture on the right shows the layers in the boxes. ① plants; ② 10 cm substrate; ③ reed; ④: filter cloth; ⑤: root barrier.

The study site was on a roof of a concrete factory in Hollola, Finland (60°59′16.86″N, 25°24′39.41″E). Constructed in September 2016, the experiment consisted of 25 boxes (0.2 × 2.1 × 1.6 m), of which 20 were used in this study. This experiment was conducted once in summer 2017. The boxes were made of plywood and walled with a plastic membrane (HD Polyethylene). The bottoms were covered with 0.5 cm filter cloth (VT Filt) and 5 cm reed (*Phragmites australis*). Ten cm substrate was added on top of the reed layer (Figure 3).

Two substrates based on lightweight crushed concrete were used in this study, one with biochar (50% concrete, 20% biochar, 25% compost, and 5% pinewood chips) and one without (70% concrete, 25% compost, and 5% pinewood chips). The biochar was produced by slow pyrolysis at 450 °C from hardwood mixtures (mainly aspen, alder, and birch) (Barbetec Oű, Pärnu, Estonia). The biochar had a water holding capacity (WHC) of 137% of its volume and a pH of 8.2. Its concentrations of C, N and P were 730 000 mg/kg, 4400 mg/kg, and 310 mg/kg, respectively. The horse manure compost (pH 6.4) was obtained from Biolan Ltd (Kauttua, Finland). It contained 15 000 mg/kg total N and 2400 mg/kg P, corresponding to 100 mg/kg soluble N and 1000 mg/kg soluble P (dry matter). Eventually, substrates with and without biochar had pH 11.6 and 11.4, 6.0% and 9.6% organic matter, 2.3 and 6.6 mg/kg soluble N, and 4.1 and 5.0 mg/kg soluble P, respectively.

The experiment had a two-factor design (substrate type and mycorrhizal inoculation) with two levels for two factors, each with five replicates. Thus, there were four different treatments: control substrate (Con), biochar amended substrate (Bc), Con amended with mycorrhiza (My), and Bc amended with mycorrhiza (BcMy) (Figure 3). MYC800 product (800 fungal spores of *R. irregularis* stain DAOM181602/g) from Lallemand Plant Care was applied to the corresponding boxes (3 g/m^2^). Forty eight seedlings of eight plant species provided by Terolan Taimitarha (8 *Thymus serpyllum*, 8 *Oreganum vulgare*, 8 *Dianthus deltoids*, 8 *F. vesca*, 4 *S. acre*, 4 *Prunella vulgaris*, 4 *Viscaria vulgaris*, and 4 *Armeria maritima*) were planted with their original substrate (6 dl peat) at 25 cm distance in each box in random order.

From April to September 2017, substrate moisture (volumetric water content) was continuously measured using Decagon 5TE sensors at 5-cm depth in the center of randomly selected three replicates of both treatments (with and without biochar). Meanwhile, rain intensity was continuously monitored using Decagon ECRN-100 rain gauge tipping bucket. The data was recorded at a 10-min resolution and stored in Decagon Em50 data loggers.

On September 6^th^ 2017, root samples of *T. serpyllum* and *F. vesca* were collected from the meadow roof as they have been reported to host *R. irregularis* (Xie et al., 2018, 2020). From each box, three random individual roots per plant species were collected, carefully washed, gently mixed, and then stored in 70% ethanol as one pooled sample. Altogether, there were 20 samples each for *T. serpyllum* and *F. vesca*.

### 2.2 Detection of *R. irregularis* and *B. amyloliquefaciens*

*R. irregularis* abundance was detected via root staining and microscopy. The root samples were stained, made into microscopic slides, and examined for AMF abundance under the microscope. In the staining process, fine root samples were transferred into a 1.5 ml Eppendorf tube filled with KOH solution. Then, the roots were transferred into H_2_O_2_ solution containing 5 ml/l NH_3_, and later HCl solution. Afterward, the root samples were held in trypan blue solution at high temperatures (Table 2). The stained roots were mounted with polyvinyl alcohol-lactic acid-glycerol solution (PVLG, 10 ml/l water, 10 ml/l lactic acid, 1 ml/l glycerol, and 1.66 mg/l polyvinyl alcohol) and made into microscopic slides. Lastly, the *R. irregularis* abundance was quantified using the gridline magnified intersection method (McGonigle et al., 1990).

**Table 2:** Detailed staining protocol for selected plant species.

Plant species	Staining solutions			
	KOH	H_2_O_2_+NH_3_^1^	HCl^2^	trypan blue^3^
*F. vesca*	48 h in 1.25% KOH at RT^4^	None	60 min at RT^4^	60 min at 80 °C
*P. alpina*	24 h in 2.5% KOH at RT^4^	120 min at RT^4^	120 min at RT^4^	60 min at 75 °C
*T. repens*	60 min in 2.5% KOH at 80 °C	None	30 min at RT^4^	90 min at 90 °C
*T. serpyllum*	20 min in 2.5% KOH at 90 °C	None	60 min at RT^4^	90 min at 80 °C
*V. tricolor*	60 min in 2.5% KOH at 80 °C	None	30 min at RT^4^	75 min at 95 °C

1 1.5% hydrogen peroxide containing 5 ml/l ammonia.

2 1% hydrochloric acid.

3 Lactic acid containing 63 ml/l glycerol, 63 ml/l water, and 0.02% trypan blue.

4 Room temperature.

*B. amyloliquefaciens* density was measured by quantifying the amount of a phylogenetic marker gene called *gyrB* in substrate DNA (Bavykin et al., 2004; Wang et al., 2007; Xie et al., 2018). The gene *gyrB* is ubiquitous in bacteria which can encode the subunit B protein of DNA gyrase. Substrate DNA was extracted from the substrate samples using the PowerSoil DNA extraction kit (MO BIO, Carlsbad, USA). Genomic DNA from the Rhizocell powder was extracted using the DNeasy Plant Mini Kit (QIAGEN, Hilden, Germany). A 94-bp *gyrB* gene fragment from the substrate/Rhizocell DNA samples was amplified by PCR with primer pair BaG3F (5′-GTCGACCACTCTTGACGTTACGGTT-3′) and BaG4R (5′-CGATCACTTCAAGATCGGCCACAG-3′). The PCR products were sequenced at Haartman Institute (Helsinki, Finland) to verify if the *Bacillus* species was the same in the substrate samples as in the Rhizocell product. Before quantifying the *gyrB* gene in the substrate DNA samples using qPCR, 1) the Rhizocell DNA sample was diluted into five series: 1:1, 1:10, 1:100, 1:1000, and 1:10000, which were used to construct a standard curve and calculate amplification efficiency; 2) substrate DNA samples were diluted to 5 ng/μl. Next, qPCR reaction followed the procedure: 5 min at 95 °C; 45 cycles of 10 s at 95 °C, 10 s at 62 °C, and 10 s at 72 °C; and 5 min at 72 °C. Finally, *Bacillus*’ densities (ng DNA/g substrate) were calculated according to Xie et al. (2018). Three subsamples were taken from each substrate sample to measure *B. amyloliquefaciens* density (3 sample replicates), producing 36 readings for each sampling time in the first experiment.

### 2.3 Statistical analysis

The abundance of hyphae, arbuscules, and vesicles underwent logit transformation. The outcomes of different treatments from each experiment were compared using the least significant difference analysis (LSD_0.05_) following analysis of variance (ANOVA). Significance levels for the effects of microbial inoculation, plant species, substrate pH, biochar amendment, and their interactions were examined by ANOVA using the SPSS software (IBM SPSS Statistics 25, Armonk, NY, USA).

Mean values of *Bacillus* density in the substrates in the first experiment were compared between treatments also using LSD_0.05_ following ANOVA. The ANOVA model included the three treatments, three sample replicates, and the plot ID. The data were tested for normality with square root transformation applied to count data.

## 3 Results and discussions

The establishment of *R. irregularis* and *B. amyloliquefaciens* in our VBE systems was successful to various degrees. In general, the PGPMs grew better in moderate rain and mild temperature (≤23 °C) during the Nordic summers. They were significantly affected by substrate pH, host plant species, and their interaction, whereas the effect of biochar amendment on *R. irregularis* did not gain strong support. In summary, our study suggests that manipulation of PGPMs in VBE systems is achievable via various factors and their interactions.

### 3.1 Weather conditions (heat and drought) influenced *B. amyloliquefaciens* density

**Figure 4:**
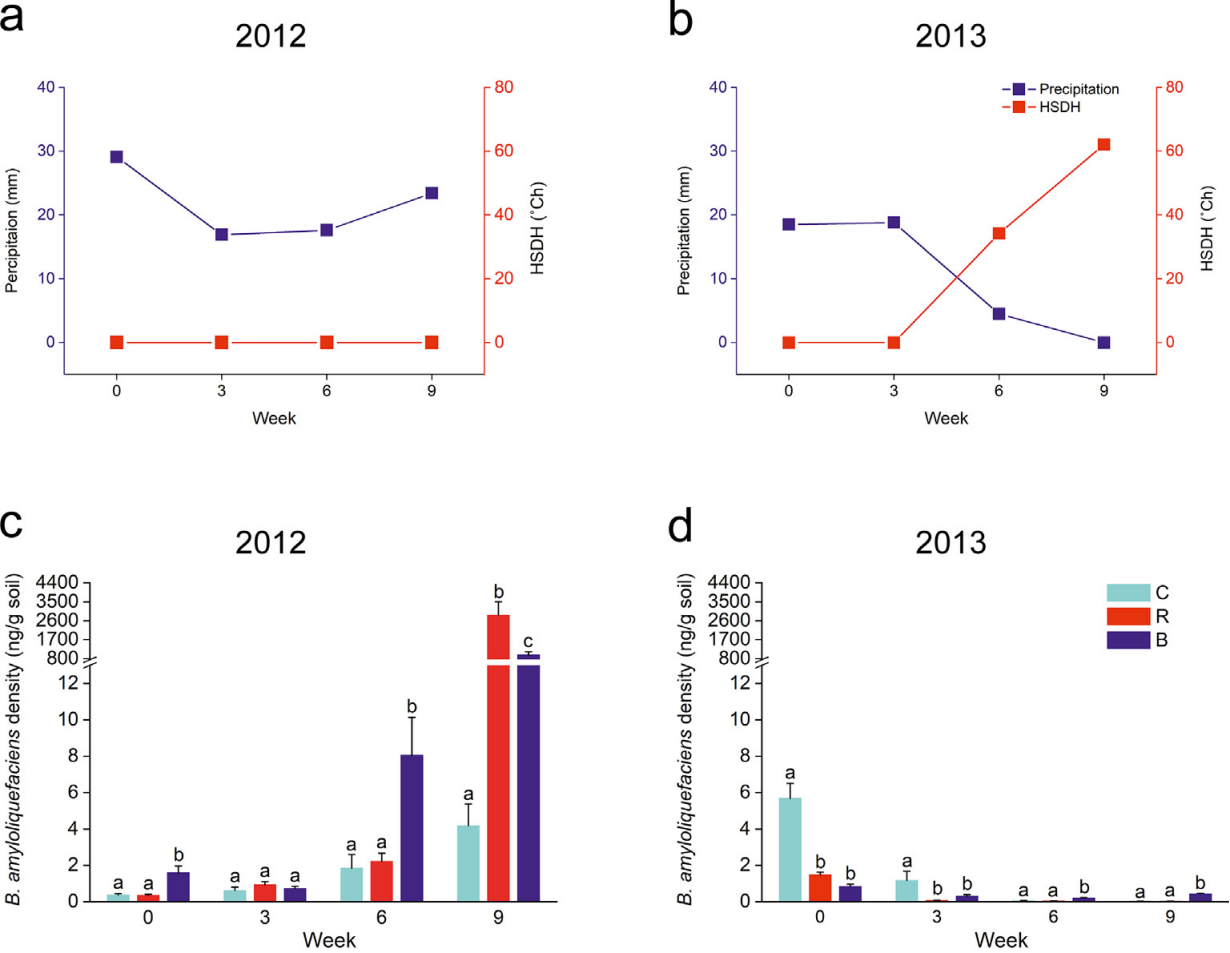
The growing conditions and *B. amyloliquefaciens* population density of each sampling time in 2012 and 2013 in the *Sedum* roof experiment. Panel a & b present precipitation and heat stress degree hour (HSDH) in 2012 (a) and 2013 (b). Panel c & d present the population density of *B. amyloliquefaciens* in each treatment and sampling in 2012 (c) and 2013 (d). Total n = 288 (3 treatments × 4 replicates × 3 sample replicates × 4 samplings × 2 years). The legends for the bars in the lower panels are C for control, R for *R. irregularis* treatment, and B for *B. amyloliquefaciens* treatment. Data are presented as mean ± SE. Different lowercase letters indicate statistical differences by LSD_0.05_.

In the *Sedum* roof experiment, weather conditions during the experimental periods in 2012 and 2013 were different, with steady precipitation between 15 and 30 mm/week and no heat stress in 2012 (Figure 4a), versus two dry periods in weeks 6 and 9 in 2013 when HSDH reached 34.2˚Ch and 62.1˚Ch, respectively (Figure 4b).

*B. amyloliquefaciens* responded to the mild weather conditions in 2012 with increased population density throughout the four measurements (Figure 4c). Compared to week 0, its population density in week 9 increased by 12 folds, 604 folds, and 8823 folds respectively for control, *B. amyloliquefaciens* treatment, and *R. irregularis* treatment. However, when the weather became dry and hot in 2013, *B. amyloliquefaciens* in all treatment plots almost disappeared in 2013 (Figure 4d).

The results suggest that the establishment of *B. amyloliquefaciens* in the maintenance-free *Sedum* roof with a thin substrate layer depended greatly on the amount of rainfall and temperature: the hotter and drier the conditions, the lower *B. amyloliquefaciens* population density in the substrate. For example, Aslim et al. (2002) found out that the optimum substrate temperature for six *Bacillus* species ranged between 28 and 37 °C, and growth ceased when the temperature exceeded 45 °C. Another study also found that under three different substrate temperatures (32, 37, and 42 °C), *B. amyloliquefaciens* DL-3 showed the highest bacterial cell concentration at 32 °C (Jo et al., 2008). In our experiment, we observed that in extreme cases in 2013, substrate temperature could reach 52 °C when the air temperature was merely 30 °C (data not shown). The high temperatures and low precipitation in 2013 likely caused an almost demise of *B. amyloliquefaciens*. *B. amyloliquefaciens* did not go extinct, but a small population persisted in the treated plots at the end of the season. When the growing conditions change or if the microbes adapt to the weather conditions, their populations may recover (Griffiths and Philippot, 2013).

Other researchers have found that low substrate moisture is detrimental to *Bacillus* species. For instance, Vardharajula et al. (2011) recorded a tenfold decrease in population density of a drought-resistant *B. amyloliquefaciens* strain HYD-B17 under 9-day drought stress with substrate moisture at 46.6% WHC, compared with 75% WHC. Thus, it is also likely that the low substrate moisture in our substrate caused the population density decline of *B. amyloliquefaciens* in 2013.

Furthermore, it has been confirmed that seasonal changes also influence bacterial communities in the substrate (Torsvik and Øvreås, 2002). For instance, freeze-thaw cycles at the turn of seasons can reduce bacterial biomass in the substrate by altering temperature, water availability, and nutrient availability (Yergeau and Kowalchuk, 2008). As a result, seasonal changes can reduce bacterial populations to a low level at which population recovery is stunted. Therefore, in our study, *B. amyloliquefaciens* inoculation in 2012 ensured a good start for bacterial growth. However, after the Nordic winter, the remaining *B. amyloliquefaciens* with low density or low viability might lead to a constrained start in 2013. If we had repeated inoculation in 2013, *B. amyloliquefaciens* would probably have exhibited a similar growth pattern as in 2012.

All the three factors mentioned above may have jointly contributed to the outcome. Further research can focus on methods that can maintain substrate moisture and temperature levels for PGPMs and improve their winter survival. Also, long-term studies are needed to reveal the impact of weather and seasonal variation on PGPMs' succession in VBE systems.

### 3.2 *R. irregularis* might promote the proliferation of *B. amyloliquefaciens*

In the *Sedum* roof experiment, the growth of *B. amyloliquefaciens* population density in *R. irregularis* treated plots equaled the one in *B. amyloliquefaciens* treated plots in week 9 in 2012, even though *B. amyloliquefaciens* was never applied in *R. irregularis* treated plots (Figure 4c). We propose that *R. irregularis* may have stimulated the growth of local *B. amyloliquefaciens* strains in the *Sedum* roof substrate. However, in a greenhouse experiment where *R. irregularis* and *B. amyloliquefaciens* were single- and co-inoculated with eight plant species, such a promoting effect was not confirmed (Xie et al., 2018). The difference between the two experiments might be the growing conditions. Plants in the greenhouse experiment were cultivated in favorable growing conditions, while *P. alpina* grew in stressed growing conditions on the *Sedum* roof.

Plants in stressed conditions (e.g. flood, drought, pathogens, and nutrient deficiency) could proactively lure beneficial microbes via root exudates containing chemoattractants, such as malic acid (Keeley, 1978; Henry et al., 2007). Malic acid has been found to stimulate the propagation of *B. subtilis* (a PGPM closely related to *B. amyloliquefaciens*) and its biofilm formation (Rudrappa et al., 2008; Chen et al., 2012). Furthermore, exudate production can be enhanced by AMF colonization (Miransari, 2011; Huang et al., 2014; Taktek et al., 2015). For instance, Ren et al. (2015) found that plants colonized with *Glomus mosseae* produced more malic acid in exudates upon disease infection by *Fusarium oxysporum* than in non-colonized control plants. The a posteriori hypothesis of the *Sedum* roof experiment is that drought and heat stresses might have elevated exudate secretion from mycorrhizal roots and/or hyphae. The exudates attracted local *B. amyloliquefaciens* strains and supported its growth to reach a high population density.

### 3.3 Substrate pH together with plant species significantly affected *R. irregularis* colonization

**Figure 5:**
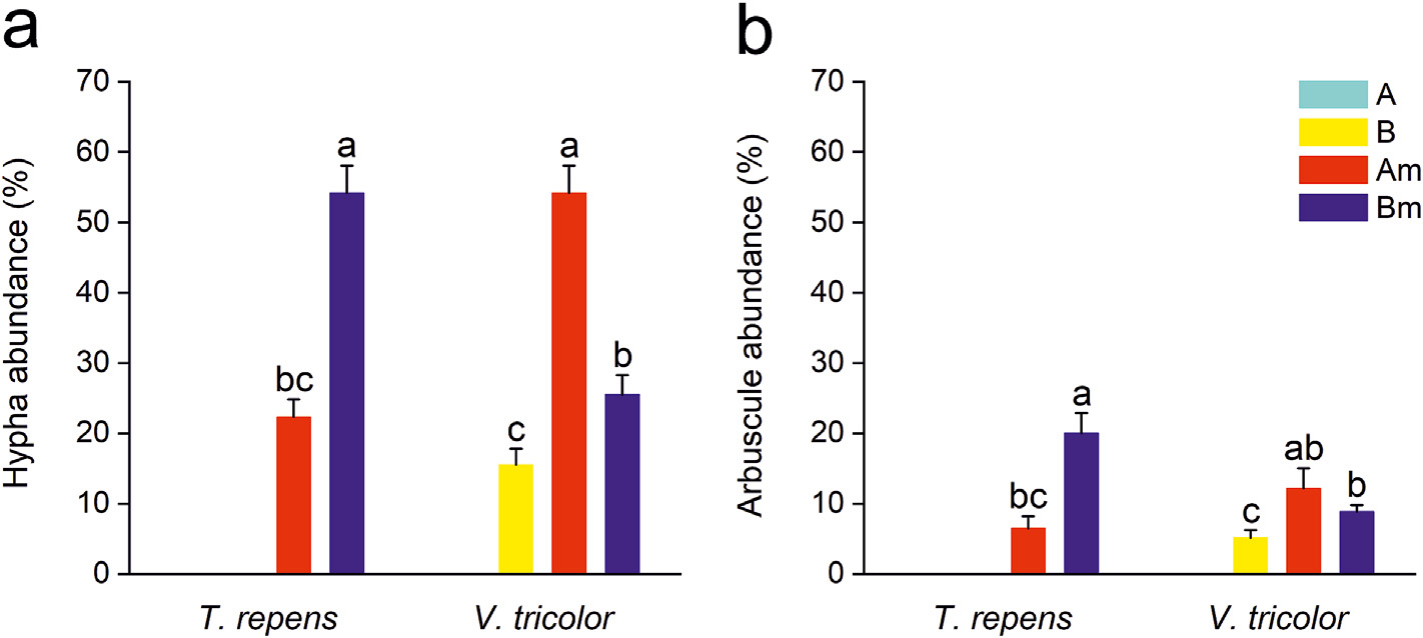
The abundance of hyphae (a) and arbuscules (b) in *T. repens* and *V. tricolor* in the balcony garden experiment. Total n = 48 (4 treatments × 2 plant species × 6 plant replicates). A: substrate A (pH 5–5.5); B: substrate B (pH 6–6.5); Am: substrate A + AMF (pH 5–5.5); Bm: substrate B + AMF (pH 6–6.5). Data are presented as mean ± SE. Different lowercase letters indicate statistical differences by LSD_0.05_.

In the balcony garden experiment, hypha and arbuscule abundance in the roots of *T. repens* and *V. tricolor* showed opposite results in response to substrate pH: *T. repens* was more colonized in the neutral substrate (pH 6-6.5), while *V. tricolor* was more colonized in the acidic substrate (pH 5-5.5) (Figure 5). Vesicles were not observed in any of the treatments and plant species. *R. irregularis* was not detected in the controls except in B substrate planted with *V. tricolor*, suggesting a trace of background AMF. Since zero values in control groups would distort ANOVA results, the controls were not included. According to the ANOVA, both hypha and arbuscule abundances were significantly affected by the interaction of plant species and substrate pH (Table 3).

**Table 3:** Effect of the substrate pH, ​plant species, and their interaction on the abundance of hyphae and arbuscules in the balcony gardens. Sp. and pH refer to plant species and substrate pH, respectively.

Source	Hypha			Arbuscule		
	df	F	Sig	df	F	Sig
Sp.	1.19	0.060	0.809	1.19	1.574	0.225
pH	1.19	0.883	0.359	1.19	4.500	<0.05
Sp.×pH	1.19	77.629	<0.01	1.19	11.988	<0.01

Our finding based on the balcony garden experiment is in line with a greenhouse study in which *Vigna unguiculata* plants were inoculated with two AMF species under three substrate pH conditions (pH 4.7, 4.9, and 5.2). *G. etunicatum* abundance was significantly improved when substrate pH shifted slightly from 4.7 to 5.2, while *Gigaspora margarita* abundance did not differ (Rohyadi et al., 2004). These findings suggest that mycorrhizal colonization is affected by substrate pH, plant species, mycorrhizal species, and their interactions. However, due to the complex interactions between substrate pH and other substrate properties indirectly affecting mycorrhizal colonization, the fundamental mechanisms remain unspecified (Leifheit et al., 2014). Therefore, we propose three plausible a posteriori hypotheses.

Firstly, substrate pH interacts with AMF colonization via reactive oxygen species (ROS) in host plants. Plants can overproduce and accumulate ROS under stresses, such as too high or too low substrate pH (Shi et al., 2006; Xia et al., 2015; Zhang et al., 2017). ROS could trigger cell death and autophagy and reduce mycorrhizal colonization to various degrees, depending on the ability of the host plant and AMF species to detoxify (Xia et al., 2015; Lenoir et al., 2016). Therefore, AMF colonization is determined by the different tolerance levels of plant and AMF species under different substrate pH conditions.

Secondly, some plant species can modify substrate pH to suitable levels. For instance, the biomass of *T. repens* decreased significantly when growing in substrate pH lower than 6.5 (Deska et al., 2011). However, *T. repens* can increase substrate pH through its root exudates as a strategy to mitigate the negative effect of substrate acidity (Snaydon, 1962). Additionally, substrate pH levels can influence phosphorous availability, and higher phosphorous content in the substrate can significantly inhibit AMF colonization (Ouzounidou et al., 2015; Klichowska et al., 2019). Therefore, we suggest that some plant species, such as *T. repens* and *V. tricolor* in this experiment, can affect AMF colonization by altering the pH and phosphorous availability in substrates.

Thirdly, the diversity and structure of AMF communities were determined by the availability of aluminum (Al) (Aguilera et al., 2017), and Al availability increases when substrate pH decreases (Driscoll and Schecher, 1990; Dong et al., 1999; Cuenca et al., 2001). Therefore, substrate pH determines Al availability to host plants which eventually affects AMF colonization. This process is also regulated by how tolerant the host plants and AMF species are towards Al (Aguilera et al., 2017).

Our findings, together with earlier ones, emphasize the complex interaction of plant species, AMF species, substrate pH, and element availability. To harness the beneficial plant-microbial symbiosis in VBE systems, we need to explore and test different situations of the above factors.

### 3.4 Biochar amendment had no effect on *R. irregularis* colonization

**Figure 6:**
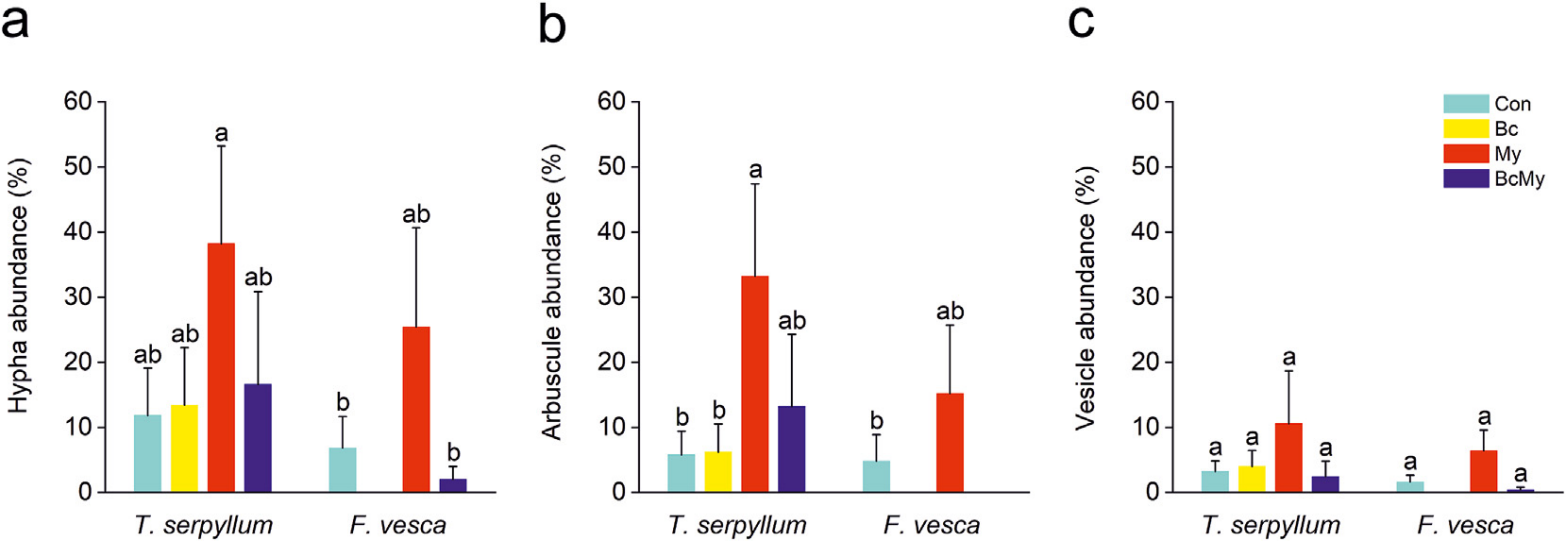
The abundance of hyphae (a), arbuscules (b), and vesicles (c) in *T. serpyllum* and *F. vesca* in the meadow roof experiment. Total n = 40 (4 treatments × 2 plant species × 5 replicates). Con: lightweight crushed concrete 70%, compost 25%, pinewood chips 5%; Bc: lightweight crushed concrete 50%, biochar 20%, compost 25%, pinewood chips 5%; My: Con with mycorrhiza; BcMy: Bc with mycorrhiza. Data are presented as mean ± SE. Different lowercase letters indicate statistical differences by LSD_0.05_.

In the meadow roof experiment, both plants were colonized by *R. irregularis*. No significant effect of biochar amendment on the mycorrhizal abundance was recorded. According to ANOVA, biochar amendment, plant species, and their interactions did not play a statistically significant role in *R. irregularis* colonization (data not shown), yet the results might suggest a negative impact rather than a positive one on mycorrhizal abundance, as BcMy treatments had lower mean abundance than My treatments for both plant species (Figure 6).

We speculate that the extremely high pH of the two substrates (11.6 and 11.4) might be the primary factor that overshadowed the effect of biochar on AMF abundance. Additionally, researchers have found biochar either beneficial, detrimental, or neutral to mycorrhizal development in plant root systems (Koide, 2017). This inconsistency may be due to differences in the properties of biochar and the many other environmental factors affecting colonization and development of mycorrhiza. Even though the exact mechanisms behind the varying outcomes are not well-established, various hypotheses have been put forth. For instance, biochar can increase phosphorus content, antagonizing AMF colonization (Nouri et al., 2014; Koide, 2017). Yet, in our experiment, the biochar amended substrates had only 0.9 mg/kg more phosphorus than those without biochar (4.1 mg/kg versus 5 mg/kg), a likely negligible difference for AMF.

**Figure 7:**
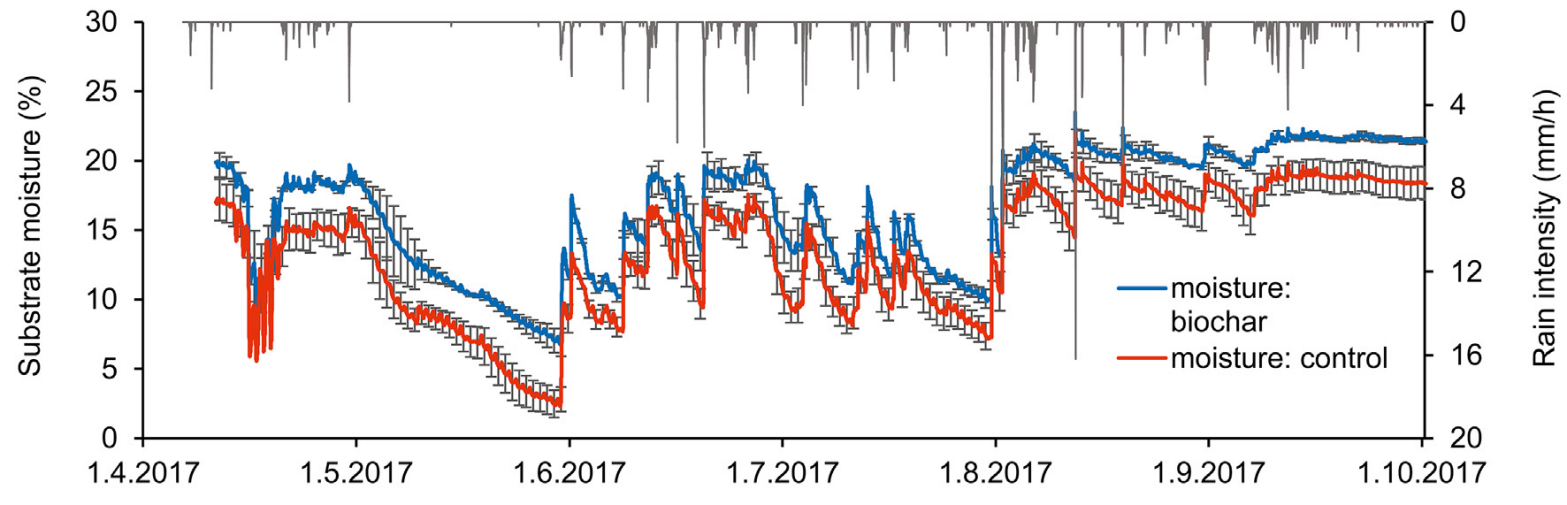
The average substrate volumetric moisture and daily rain intensity in the treatments with and without biochar from April to September 2017 in the meadow roof experiment. Data of substrate volumetric moisture are presented as mean ± SE.

As a highly porous material, biochar can increase WHC to provide a suitable habitat for substrate microbes (Warnock et al., 2007). We also confirmed in our meadow roof experiment that vegetated boxes amended with biochar (Bc and BcMy) had mostly 2–5% higher moisture content than control boxes (Con and My), except during the rainy mid-summer (June–July) period (Figure 7). Since most of the growing season in 2017 was rather rainy, the conditions were not critical for mycorrhiza in either treatment regarding water availability, which is likely to be one of the reasons for negligible differences in AMF development between the treatments. In addition, biochar amendment can influence AMF colonization by inducing microbial interaction, such as attracting mycorrhizal helper bacteria and phosphate solubilizing bacteria (Pietikäinen et al., 2000; Warnock et al., 2007). These mechanisms could have both positive and negative effects on mycorrhizal colonization, and altogether contribute to the AMF abundance (Warnock et al., 2007).

### 3.5 *R. irregularis* exhibited variable colonization efficiency towards different plant species in both field and lab conditions

We illustrated that AMF can survive and establish on vegetated roofs and facades, which complies with the findings from Rumble et al. (2018). *F. vesca*, *T. repens*, *T. serpyllum*, and *V. tricolor* showed various colonization levels by *R. irregularis* in different treatments. In contrast, *P. alpina* was not colonized by *R. irregularis* either on the vegetated roof or in the lab (data not shown), suggesting that *P. alpina* might not be a suitable host for *R. irregularis*, although it has been reported as a known mycorrhizal plant (Cripps and Eddington, 2005). *P. alpina* is a stress-resistant plant species that may not need mycorrhizal colonization for growth promotion (Mao and Huff, 2012; Steiner et al., 2012; Pecetti et al., 2015). By not forming mycorrhizal symbiosis, *P. alpina* could save 4–20% of photosynthates, which is normally transferred to the symbiont (Wright et al., 1998). Gange et al. (1999) even recorded a negative correlation between the abundance of AMF and a *Poa* species named *P. annua*.

The relation between host plant species and AMF compatibility has been revealed before (Molina and Horton, 2015; Xie et al., 2018). Sanders (2003) suggested that AMF symbiosis is not a species-specific interaction, meaning a given AMF species can colonize a group of plant species, and a given plant species can be colonized by different AMF species. This mechanism ensures a higher chance of mycorrhizal colonization, which benefits both the AMF and plants. However, AMF abundance is dependent on the attractiveness of the plant root exudates towards AMF species (Legay et al., 2016; Maclean et al., 2017). Therefore, we suggest testing the potential combinations of plant and AMF species in both controlled and field conditions to verify the mechanism(s) and the functionality in VBE systems (Xie et al., 2018, 2020).

## 4 Conclusions

**Figure 8:**
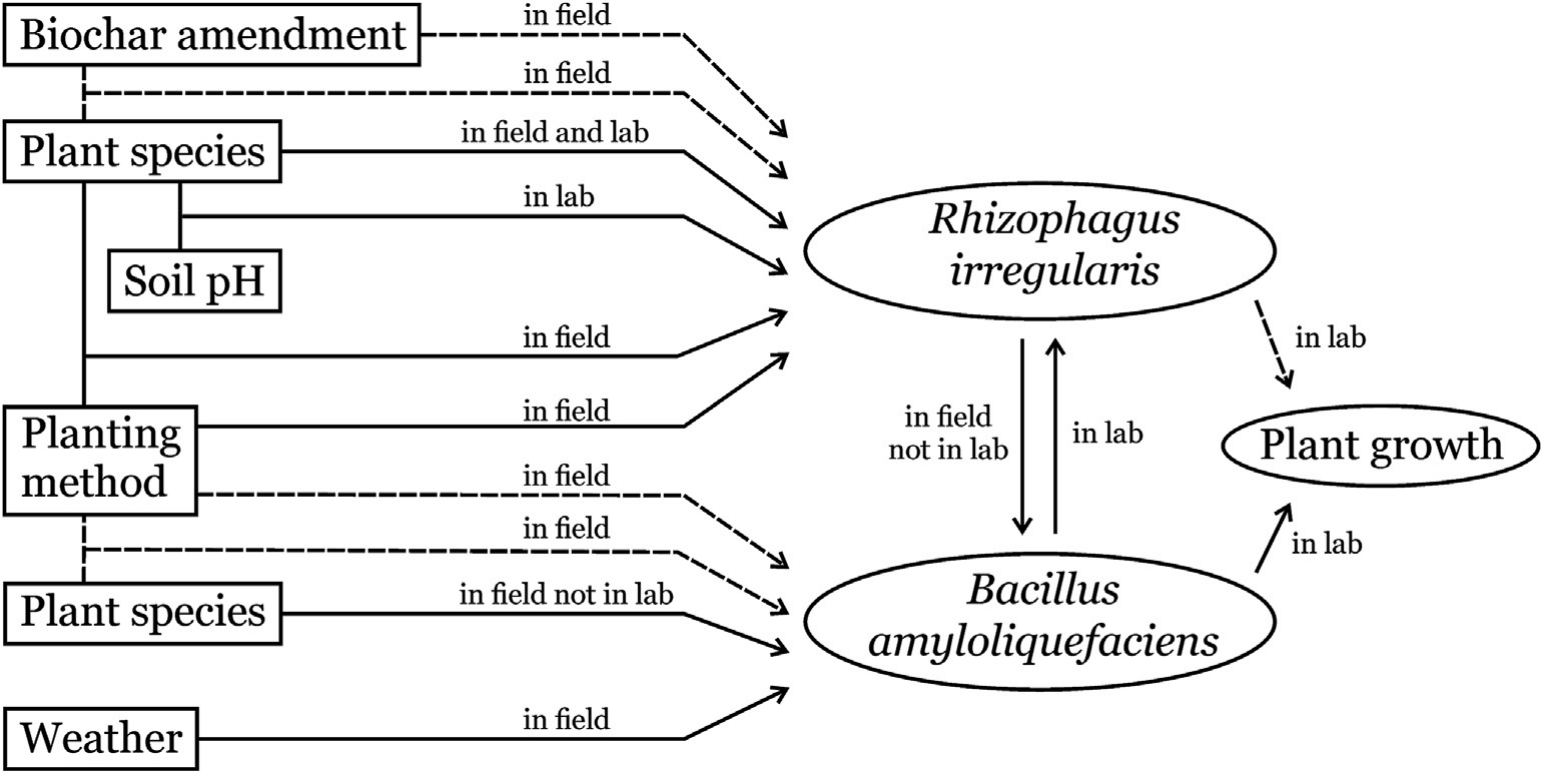
Effects of weather, plant species, biochar amendment, planting method, substrate pH, and their interactions on the microbial population of *R. irregularis* and *B. amyloliquefaciens* from previous and present research, adapted from Xie (2020). A solid arrow indicates an effect detected. A dashed arrow indicates no statistically significant effect.

According to the three experiments, the survival of *R. irregularis* and *B. amyloliquefaciens* was confirmed in vegetated roofs and facades. We conclude that: 1) Heat and drought negatively affected the population density of *B. amyloliquefaciens*, making it an unreliable growth-promoting microbe in maintenance-free (no irrigation scheme) vegetated roofs with thin substrate layers; 2) *R. irregularis* might support the growth of *B. amyloliquefaciens* under harsh conditions; 3) *R. irregularis* abundance was influenced by the interaction of substrate pH and plant species; and 4) The impact of biochar amendment on *R. irregularis* colonization exhibited a negative effect rather than a positive one, which did not comply with our hypothesis. It was probably due to the extremely high alkalinity of the concrete-based substrate obfuscating the effect of biochar. Thus, we suggest further studies on AMF in less alkaline substrates amended with biochar. Based on previous and present related research, we propose a map indicating the effects of various factors and their interactions on the microbial population of the PGPMs and plant growth based on VBE studies (Figure 8).

While our experiments confirmed the possibility of inoculating PGPMs in substrates of VBEs, we recommend moderate irrigation on VBEs when prolonged dry and hot weather occurs to help plants and substrate microbes survive. However, irrigation should be minimal to allow them to adapt to dry and hot conditions. Substrate pH should be adjusted according to VBE plant species to achieve successful mycorrhizal colonization. Although *B. amyloliquefaciens* seemed unreliable in our study, we could still maintain and accommodate the beneficial microbe to the local weather conditions via repeated inoculation. Pre-grown plants can be inoculated with PGPMs before transplanting to VBE systems, ensuring successful colonization and sparing the effort of on-site inoculation. The usefulness of biochar amendment on VBEs should be further tested with a wide range of substrate pH, host plant species, and PGPM species. Moreover, the possible promoting effect of *R. irregularis* on *B. amyloliquefaciens* in field conditions needs further verification. If proved correct, co-inoculation of *R. irregularis* and *B. amyloliquefaciens* in VBEs could be an effective practice to support plant growth under such suboptimal growing conditions.

## Declarations

### Author contribution statement

Long Xie: Conceived and designed the experiments; Performed the experiments; Analyzed and interpreted the data; Contributed reagents, materials, analysis tools or data; Wrote the paper.

Sari Timonen; Susanna Lehvävirta: Conceived and designed the experiments; Analyzed and interpreted the data; Contributed reagents, materials, analysis tools or data.

Alan C. Gange: Analyzed and interpreted the data.

Kirsi Kuoppamäki; Marleena Hagner: Contributed reagents, materials, analysis tools or data; Wrote the paper.

### Funding statement

This work was supported by China Scholarship Council (grant number 20140796003), Maiju and Yrjö Rikala Horticultural Foundation, Finnish Cultural Foundation (grant number 00161123), and Kone Foundation (grant number 201608812). Open access is funded by Helsinki University Library.

### Data availability statement

Data will be made available on request.

### Declaration of interests statement

The authors declare no conflict of interest.

### Additional information

No additional information is available for this paper.

## References

[bib1] Aguilera P., Marín C., Oehl F., Godoy R., Borie F., Cornejo P. (2017). Selection of aluminum tolerant cereal genotypes strongly influences the arbuscular mycorrhizal fungal communities in an acidic Andosol. Agric. Ecosyst. Environ..

[bib2] Anderson C.R., Condron L.M., Clough T.J., Fiers M., Stewart A., Hill R.A., Sherlock R.R. (2011). Biochar induced soil microbial community change: implications for biogeochemical cycling of carbon, nitrogen and phosphorus. Pedobiologia.

[bib3] Aslim B., Sağlam N., Beyatli Y. (2002). Determination of some properties of Bacillus isolated from soil. Turkish J. Biol..

[bib4] Barlow K.M., Mortensen D.A., Drohan P.J. (2020). Soil pH influences patterns of plant community composition after restoration with native-based seed mixes. Restor. Ecol..

[bib5] Bavykin S.G., Lysov Y.P., Zakhariev V., Kelly J.J., Jackman J., Stahl D.A., Cherni A. (2004). Use of 16S rRNA, 23S rRNA, and gyrB gene sequence analysis to determine phylogenetic relationships of Bacillus cereus group microorganisms. J. Clin. Microbiol..

[bib6] Cao C.T.N., Farrell C., Kristiansen P.E., Rayner J.P. (2014). Biochar makes green roof substrates lighter and improves water supply to plants. Ecol. Eng..

[bib7] Chen Y., Cao S., Chai Y., Clardy J., Kolter R., Guo J.H., Losick R. (2012). A Bacillus subtilis sensor kinase involved in triggering biofilm formation on the roots of tomato plants. Mol. Microbiol..

[bib8] Columbia Green Technologies (2014). https://columbia-green.com/wp-content/uploads/2014/08/CGT_Maintenance_July2014.pdf.

[bib9] Cripps C.L., Eddington L.H. (2005). Distribution of mycorrhizal types among alpine vascular plant families on the Beartooth Plateau, Rocky Mountains, U.S.A., in reference to large-scale patterns in arctic-alpine habitats. Arctic Antarct. Alpine Res..

[bib10] Cuenca G., De Andrade Z., Meneses E. (2001). The presence of aluminum in arbuscular mycorrhizas of Clusia multiflora exposed to increased acidity. Plant Soil.

[bib11] Deska J., Jankowski K., Bombik A., Jankowska J. (2011). Effect of growing medium pH on germination and initial development of some grassland plants. Acta Sci. Pol. Agric..

[bib12] Dong D., Xie Z., Du Y., Liu C., Wang S. (1999). Influence of soil pH on aluminum availability in the soil and aluminum in tea leaves. Commun. Soil Sci. Plant Anal..

[bib13] Driscoll C.T., Schecher W.D. (1990). The chemistry of aluminum in the environment. Environ. Geochem. Health.

[bib14] Fulthorpe R., MacIvor J.S., Jia P., Yasui S.L.E. (2018). The green roof microbiome: Improving plant survival for ecosystem service delivery. Front. Ecol. Evol..

[bib15] Gange A.C., Lindsay D.E., Ellis L.S. (1999). Can arbuscular mycorrhizal fungi be used to control the undesirable grass Poa annua on golf courses?. J. Appl. Ecol..

[bib16] Griffiths B.S., Philippot L. (2013). Insights into the resistance and resilience of the soil microbial community. FEMS Microbiol. Rev..

[bib17] Gu S. (2016). Growing degree hours - a simple, accurate, and precise protocol to approximate growing heat summation for grapevines. Int. J. Biometeorol..

[bib18] Henry A., Doucette W., Norton J., Bugbee B. (2007). Changes in crested wheatgrass root exudation caused by flood, drought, and nutrient stress. J. Environ. Qual..

[bib19] Henry A., Frascaria-Lacoste N. (2012). The green roof dilemma - discussion of Francis and Lorimer (2011). J. Environ. Manag..

[bib20] Hoch J.M.K., Rhodes M.E., Shek K.L., Dinwiddie D., Hiebert T.C., Gill A.S., Estrada A.E.S., Griffin K.L., Palmer M.I., McGuire K.L. (2019). Soil microbial assemblages are linked to plant community composition and contribute to ecosystem services on urban green roofs. Front. Ecol. Evol..

[bib21] Huang X.F., Chaparro J.M., Reardon K.F., Zhang R., Shen Q., Vivanco J.M. (2014). Rhizosphere interactions: root exudates, microbes, and microbial communities1. Botany.

[bib22] Idriss E.E., Makarewicz O., Farouk A., Rosner K., Greiner R., Bochow H., Richter T., Borriss R. (2002). Extracellular phytase activity of Bacillus amyloliquefaciens FZB45 contributes to its plant-growth-promoting effect. Microbiology.

[bib23] Jo K.I., Lee Y.J., Kim B.K., Lee B.H., Chung C.H., Nam S.W., Kim S.K., Lee J.W. (2008). Pilot-scale production of carboxymethylcellulase from rice hull by Bacillus amyloliquefaciens DL-3. Biotechnol. Bioproc. Eng..

[bib24] Keeley J.E. (1978). Malic acid accumulation in roots in response to flooding: Evidence contrary to its role as an alternative to ethanol. J. Exp. Bot..

[bib25] Klichowska E., Nobis M., Piszczek P., Błaszkowski J., Zubek S. (2019). Soil properties rather than topography, climatic conditions, and vegetation type shape AMF–feathergrass relationship in semi-natural European grasslands. Appl. Soil Ecol..

[bib26] Koide R.T., Johnson N.C., Gehring C., Jansa J. (2017). Mycorrhizal Mediation of Soil: Fertility, Structure, and Carbon Storage.

[bib27] Kuoppamäki K., Hagner M., Lehvävirta S., Setälä H. (2016). Biochar amendment in the green roof substrate affects runoff quality and quantity. Ecol. Eng..

[bib28] Kuoppamäki K., Hagner M., Valtanen M., Setälä H. (2019). Using biochar to purify runoff in road verges of urbanised watersheds: A large-scale field lysimeter study. Watershed Ecol. Environ..

[bib29] Kuoppamäki K., Lehvävirta S. (2016). Mitigating nutrient leaching from green roofs with biochar. Landsc. Urban Plann..

[bib30] Legay N., Grassein F., Binet M.N., Arnoldi C., Personeni E., Perigon S., Poly F., Pommier T., Puissant J., Clément J.C., Lavorel S., Mouhamadou B. (2016). Plant species identities and fertilization influence on arbuscular mycorrhizal fungal colonisation and soil bacterial activities. Appl. Soil Ecol..

[bib31] Leifheit E.F., Veresoglou S.D., Lehmann A., Morris E.K., Rillig M.C. (2014). Multiple factors influence the role of arbuscular mycorrhizal fungi in soil aggregation-a meta-analysis. Plant Soil.

[bib32] Lenoir I., Fontaine J., Lounès-Hadj Sahraoui A. (2016). Arbuscular mycorrhizal fungal responses to abiotic stresses: A review. Phytochemistry.

[bib33] Maclean A.M., Bravo A., Harrison M.J. (2017). Plant signaling and metabolic pathways enabling arbuscular mycorrhizal symbiosis. Plant Cell.

[bib34] Mäkelä-Kurtto R., Sippola J. (2002). Monitoring of Finnish arable land: changes in soil quality between 1987 and 1998. Agric. Food Sci. Finl..

[bib35] Mao Q., Huff D.R. (2012). The evolutionary origin of Poa annua L. Crop Sci..

[bib36] McGonigle T.P., Miller M.H., Evans D.G., Fairchild G.L., Swan J.A. (1990). A new method which gives an objective measure of colonization of roots by vesicular—arbuscular mycorrhizal fungi. New Phytol..

[bib37] McGuire K.L., Payne S.G., Palmer M.I., Gillikin C.M., Keefe D., Kim S.J., Gedallovich S.M., Discenza J., Rangamannar R., Koshner J.A., Massmann A.L., Orazi G., Essene A., Leff J.W., Fierer N. (2013). Digging the New York City skyline: soil fugal communities in green roofs and city parksn. PLoS One.

[bib38] Miransari M. (2011). Interactions between arbuscular mycorrhizal fungi and soil bacteria. Appl. Microbiol. Biotechnol..

[bib39] Molina R., Horton T.R., Horton T.R. (2015). Mycorrhizal Networks.

[bib40] Molineux C.J., Connop S.P., Gange A.C. (2014). Manipulating soil microbial communities in extensive green roof substrates. Sci. Total Environ..

[bib41] Molineux C.J., Gange A.C., Newport D.J. (2017). Using soil microbial inoculations to enhance substrate performance on extensive green roofs. Sci. Total Environ..

[bib42] Nouri E., Breuillin-Sessoms F., Feller U., Reinhardt D. (2014). Phosphorus and nitrogen regulate arbuscular mycorrhizal symbiosis in petunia hybrida. PLoS One.

[bib43] Ouzounidou G., Skiada V., Papadopoulou K.K., Stamatis N., Kavvadias V., Eleftheriadis E., Gaitis F. (2015). Effects of soil pH and arbuscular mycorrhiza (AM) inoculation on growth and chemical composition of chia (Salvia hispanica L.) leaves. Rev. Bras. Bot..

[bib44] Palansooriya K.N., Wong J.T.F., Hashimoto Y., Huang L., Rinklebe J., Chang S.X., Bolan N., Wang H., Ok Y.S. (2019). Response of microbial communities to biochar-amended soils: a critical review. Biochar.

[bib45] Pecetti L., Romani M., Spoleto P., Tosca A., Della Marianna G., Gusmeroli F. (2015). Morpho-physiological variation of Poa alpina L. genetic resources from the Rhaetian Alps, Italy, grown in two altitude-contrasting sites. Grass Forage Sci..

[bib46] Pietikäinen J., Kiikkilä O., Fritze H. (2000). Charcoal as a habitat for microbes and its effect on the microbial community of the underlying humus. Oikos.

[bib47] Rayner J.P., Farrell C., Raynor K.J., Murphy S.M., Williams N.S.G. (2016). Plant establishment on a green roof under extreme hot and dry conditions: The importance of leaf succulence in plant selection. Urban For. Urban Green..

[bib48] Ren L., Zhang N., Wu P., Huo H., Xu G., Wu G. (2015). Arbuscular mycorrhizal colonization alleviates Fusarium wilt in watermelon and modulates the composition of root exudates. Plant Growth Regul..

[bib49] Rohyadi A., Smith F.A., Murray R.S., Smith S.E. (2004). Effects of pH on mycorrhizal colonisation and nutrient uptake in cowpea under conditions that minimise confounding effects of elevated available aluminium. Plant Soil.

[bib50] Rudrappa T., Czymmek K.J., Paré P.W., Bais H.P. (2008). Root-secreted malic acid recruits beneficial soil bacteria. Plant Physiol..

[bib51] Rumble H., Finch P., Gange A.C. (2018). Green roof soil organisms: anthropogenic assemblages or natural communities?. Appl. Soil Ecol..

[bib52] Rumble H., Gange A.C. (2017). Microbial inoculants as a soil remediation tool for extensive green roofs. Ecol. Eng..

[bib53] Sanders I.R. (2003). Preference, specificity and cheating in the arbuscular mycorrhizal symbiosis. Trends Plant Sci..

[bib54] Shafique M., Kim R., Rafiq M. (2018). Green roof benefits, opportunities and challenges – A review. Renew. Sustain. Energy Rev..

[bib55] Shi Q., Zhu Z., Li J., Qian Q. (2006). Combined effects of excess Mn and low pH on oxidative stress and antioxidant enzymes in cucumber roots. Agric. Sci. China.

[bib56] Snaydon R.W. (1962). Micro-distribution of Trifolium repens L. and its relation to soil factors. J. Ecol..

[bib57] Starr M., Westman C.J., Ala-Reini J. (1996). The acid buffer capacity of some Finnish forest soils: Results of acid addition laboratory experiments. Water. Air. Soil Pollut..

[bib58] Steiner B.L., Armbruster G.F.J., Scheepens J.F., Stöcklin J. (2012). Distribution of bulbil- and seed-producing plants of Poa alpina (Poaceae) and their growth and reproduction in common gardens suggest adaptation to different elevations. Am. J. Bot..

[bib59] Strack D., Fester T., Hause B., Schliemann W., Walter M.H. (2003). Arbuscular mycorrhiza: biological, chemical, and molecular aspects. J. Chem. Ecol..

[bib60] Taktek S., Trépanier M., Servin P.M., St-Arnaud M., Piché Y., Fortin J.A., Antoun H. (2015). Trapping of phosphate solubilizing bacteria on hyphae of the arbuscular mycorrhizal fungus Rhizophagus irregularis DAOM 197198. Soil Biol. Biochem..

[bib61] Torsvik V., Øvreås L. (2002). Microbial diversity and function in soil: From genes to ecosystems. Curr. Opin. Microbiol..

[bib62] Vardharajula S., Ali S.Z., Grover M., Reddy G., Bandi V. (2011). Drought-tolerant plant growth promoting bacillus spp.: Effect on growth,osmol ytes,and antioxidant status of maize under drought stress. J. Plant Interact..

[bib63] Wang H., Qin J., Hu Y. (2017). Are green roofs a source or sink of runoff pollutants?. Ecol. Eng..

[bib64] Wang L.T., Lee F.L., Tai C.J., Kasai H. (2007). Comparison of gyrB gene sequences, 16S rRNA gene sequences and DNA-DNA hybridization in the Bacillus subtilis group. Int. J. Syst. Evol. Microbiol..

[bib65] Warnock D.D., Lehmann J., Kuyper T.W., Rillig M.C. (2007). Mycorrhizal responses to biochar in soil - Concepts and mechanisms. Plant Soil.

[bib66] Wright D.P., Read D.J., Scholes J.D. (1998). Mycorrhizal sink strength influences whole plant carbon balance of Trifolium repens L. Plant. Cell Environ..

[bib67] Xia X.J., Zhou Y.H., Shi K., Zhou J., Foyer C.H., Yu J.Q. (2015). Interplay between reactive oxygen species and hormones in the control of plant development and stress tolerance. J. Exp. Bot..

[bib68] Xie L. (2020).

[bib69] Xie L., Lehvävirta S., Timonen S., Kasurinen J., Niemikapee J., Valkonen J.P.T. (2018). Species-specific synergistic effects of two plant growth—Promoting microbes on green roof plant biomass and photosynthetic efficiency. PLoS One.

[bib70] Xie L., Lehvävirta S., Valkonen J.P.T. (2020). Case study: Planting methods and beneficial substrate microbes effect on the growth of vegetated roof plants in Finland. Urban For. Urban Green..

[bib71] Yergeau E., Kowalchuk G.A. (2008). Responses of Antarctic soil microbial communities and associated functions to temperature and freeze-thaw cycle frequency. Environ. Microbiol..

[bib72] Zhang H., Liu X.L., Zhang R.X., Yuan H.Y., Wang M.M., Yang H.Y., Ma H.Y., Liu D., Jiang C.J., Liang Z.W. (2017). Root damage under alkaline stress is associated with reactive oxygen species accumulation in rice (Oryza sativa L.). Front. Plant Sci..

